# Direct and Indirect Effects of Business Environment on BRI Countries’ Global Value Chain Upgrading

**DOI:** 10.3390/ijerph182312492

**Published:** 2021-11-27

**Authors:** Shengbing He, Huilin Yao, Zhou Ji

**Affiliations:** 1 School of Business, Hunan University of Science and Technology, Xiangtan 411201, China; heshengbing@163.com (S.H.); 190115010002@mail.hnust.edu.cn (H.Y.); 2 Shanghai National Accounting Institute, Shanghai 201702, China

**Keywords:** business environment, FDI, BRI countries, status elevation the global value chain

## Abstract

This study incorporates business environment, foreign direct investment (FDI), and the global value chain upgrading into a unified analysis framework to unravel the effects of business environment and FDI on the Belt and Road Initiative (BRI) countries’ status elevation on the global value chain. The panel data of 112 BRI countries from 2007 to 2017 are employed for empirical tests on the trilateral relationship through the panel data regression model. The results show: (1) business environment improvement and FDI inflow significantly promote BRI countries’ status elevation on the global value chain. Business environment not only elevates BRI countries’ status on the global value chain directly, but indirectly lifts their status through the intermediate effects of FDI; (2) business environment and FDI significantly promote the status elevation on the global value chain for industries that are intensive on varied factors, especially for labor-intensive industry; (3) the test results of the panel threshold model further verify the positive effect of the business environment and FDI inflows on BRI countries’ status elevation on the global value chain.

## 1. Introduction

In recent years, as trade liberalization deepens and technological innovation progresses, an increasing number of countries participate in the division of labor on the global value chain based on their own economic development, factor variety, and resource endowment. In September 2013, Chinese President Xi Jinping coined the Belt and Road Initiative and incorporated the countries involved in the Belt and Road Initiative (hereinafter referred to as BRI countries) into a regional economic cooperation framework characterized by consultation, contribution, and shared benefits, pushing forward new progress in economic globalization. Due to the difference in product advantages of BRI countries, their status, and roles in the division of labor on the global value chain are also varied. Developed countries dominate the high-end section on the global value chain thanks to their leading technologies and essential roles on key links. However, the majority of BRI countries are developing countries with relatively low level of economic development. The products which they produce or export contain little technology, locking themselves in the low end on the global value chain. Furthermore, Asian countries are losing their advantages in product costs, and developed countries are implementing reindustrialization and reshoring manufacturing strategies. BRI countries are therefore facing the dual challenges of backflow of high-end industries and outflow of low-end industries. It has become a focus of BRI countries to resolve their bottlenecks in manufacturing, to improve their competitiveness in international division of labor, and ultimately to move up to the medium and high range on the global value chain.

As research on the global value chain digs deeper, scholars from China and other countries narrow their interest on the factors affecting division of labor on the global value chain. Specifically, the factors affecting division of labor on the global value chain include environmental regulation [1,2], factor endowment [3,4], human capital [5], R&D intensity [6,7], industrial agglomeration [8], financing restriction [9], and business environment etc. Under current complex and changeable domestic situation, the State Council of China emphasizes that improvement of business environment is more important than preferential policies granted by governments at all levels for the development of manufacturing industry. Business environment refers to the total sum of factors and conditions which affects, to some extent, the behavior of market entities within the region, including factors such as political environment, economic and market environment, legal environment, financing environment, and social service environment. Studies on the role of business environment draw a unanimous conclusion that business environment is the key factor in economic development of countries and exerts significant impacts on sustained growth in economic competitiveness [10]. The reason for the importance of business environment is that favorable business environment effectively lowers transaction costs for companies and helps companies explore the international market [11]. Researchers believe business environment affects the efficiency of contract enforcement by companies in a country, making itself a vital factor impacting the country’s integration into the division of labor on the global value chain [12]. System environment is an essential part of business environment. Supportive system environment helps increase the sophistication level of export technologies, contributing to status elevation on the global value chain of the country [13]. Improvement of system significantly elevates the status in the division of labor on the global value chain. Favorable system environment is beneficial to the improvement of technology level and product quality. Unleashing system dividend is a vital strategic choice for China to move up on the global value chain [13]. In particular, the improvement of economic system significantly propels economic growth in BRI countries and affects their participation and status in division of labor on the global value chain [14]. Dai (2020) investigates the relation between business environment and division of labor on the global value chain [15]. He reveals improvement of business environment delivers positive effects on improvement and elevation of status in division of labor on the global value chain. In fact, foreign direct investment also plays a vital role in status elevation on the global value chain [16]. Although significantly promoting high-quality growth of the Chinese economy [17], FDI also affects the status of China’s manufacturing industry on the global value chain through technological advancement, import of intermediate goods, and embedment into the global value chain [18,19]. FDI restrains the influence of industrial conglomeration on status elevation for manufacturing industry on the global value chain. Research results indicate the influence of industrial conglomeration on manufacturing status elevation on the global value chain is different due to varied levels of FDI. When FDI is below the threshold value, industrial conglomeration suppresses status elevation of manufacturing industry on the global value chain. However, when FDI surpasses the threshold value, industrial conglomeration significantly elevates manufacturing industry’s status on the global value chain. However, business environment, FDI, and global value chain are seldom put into a unified framework for comprehensive consideration. Do business environment and FDI affect BRI countries’ status elevation on the global value chain? If yes, what is the mechanism through which business environment and FDI affect BRI countries’ status elevation on the global value chain? Is there a heterogeneity in the impacts of business environment and FDI due to different factor intensity among industries?

To unravel the above questions, this study put business environment, FDI, and status elevation on the global value chain under a unified analysis framework. From a theoretical and empirical perspective, this study examines the impact of business environment and FDI on BRI countries’ status elevation on the global value chain. Compared with existing research, this study contributes in the following aspects: (1) against the backdrop of the Belt and Road Initiative, this study focuses on business environment to investigate its direct impacts on status elevation on the global value chain, which serves as a supplement to current research; (2) combing FDI’s role in status elevation on the global value chain, this study employs intermediate effect model to explore the indirect effect of business environment on status elevation on the global value chain. It is a new way to illustrate the mechanism through which business environment affects countries’ status on the global value chain; (3) this study uses threshold effects to determine the tipping point of the impact of business environment improvement and FDI inflow on countries’ status elevation on the global value chain. Industry-specific heterogeneity is also investigated in an in-depth manner. Such practice not only enriches the study on factors affecting countries’ status on the global value chain but offers policy suggestions on elevating BRI countries’ status on the global value chain by improving business environment.

## 2. Theoretical Analysis and Hypothesis

### 2.1. Direct Effect of Business Environment on Status Elevation on the Global Value Chain

Business environment is the breeding ground for market economy and is the life-giving oxygen for market entities. Only by further improving business environment can productivity be emancipated, and competitiveness enhanced. Improvement in business environment significantly increases domestic value added in gross exports [20]. Investment facilitation helps improve total factor productivity in BRI countries [21,22]. Enhancing companies’ innovation capability is the most important in lifting the status in international division of labor on the global value chain, and improvement of business environment stimulates innovation vitality [23,24]. Innovation activities require motivation and basic system support from desirable innovation environment to lower innovation costs [25]. Desirable business environment decreases, on the one hand, the cost of starting a business, offering convenience and support for market entry or exit by companies. On the other hand, it also lowers the operation costs of companies [26]. The transaction cost and system cost are also eased, leaving companies unshackled for technological innovation. Humphrey (2014) points out technological advancement is the key factor in elevating the status on the global value chain [27]. As companies continue to innovate technologies, improve product quality, and upgrade product function, products gain stronger competitiveness at the market, leading to status elevation on the global value chain. Therefore, this study proposes the following hypothesis:

**Hypothesis** **1.** *Improvement in business environment elevates BRI countries’ status on the global value chain*.

### 2.2. FDI’s Impact on the Host Country’s Status Elevation on the Global Value Chain

FDI plays a vital role in companies’ participation in the global value chain [28] and technological progress [17]. For BRI countries to move up on the global value chain, the most important way lies in promotion of technological progress. FDI helps the host country climb up the global value chain through the following three mechanisms. First, FDI inflow increases the value added of domestically produced products for export. FDI inflow offers capital for production at the host country and promotes expansion of production scale, which attracts more multinationals to provide quality intermediate input products and supporting services, leading to increase of value added of domestically produced products for export. Meanwhile, the intermediate product brought by FDI inflow increases the production supply by local upstream companies, which expands export scale of domestic companies, improves product use efficiency, and makes companies further embedded into the global value chain. FDI increases domestic value added in gross export by attracting inflow of intermediate products and embedment into the global value chain [19]. Second, FDI inflow significantly promotes product quality improvement. FDI inflow generates demonstration effects on companies in the host country and drives local companies to improve and innovate by drawing from production mode and management model of foreign-invested companies based on their own conditions. The productivity efficiency at local companies is therefore improved. The methods and models proven effective for local companies help them move up on the global value chain. Third, FDI inflow effectively facilitates technological innovation. Continuous FDI inflow brings about fierce competition for companies at the host country and urges them to make innovation and breakthrough in key technologies, which becomes the driving forces for technological innovation at local companies. To win the competition with foreign companies, local companies must strengthen technological learning and innovation by increasing R&D input. As a result, technological level of the host country is elevated, and productivity and export product quality are improved, leading to status elevation on the global value chain.

**Hypothesis** **2.** *FDI inflow elevates BRI countries’ status on the global value chain*.

### 2.3. Indirect Effect of Business Environment on BRI Countries’ Status Elevation on the Global Value Chain

Creating favorable business environment is one of the vital factors in maintaining fast and sustained economic growth. We believe business environment affects countries’ status elevation on the global value chain through FDI by the following two channels. First, a country can effectively lower the entry costs and operation risks for FDI by optimizing market and business environment such as renovating infrastructure, enhancing system transparency and bettering financial service system [21]. In such cases, FDI can be channeled into the host country at a rapid pace and stimulate market competition and innovation. Specifically, improvement in business environment at the host country brings down barriers preventing FDI from entering, which shortens the time needed for FDI to access the market and lowers the entry costs and risks for foreign-invested companies substantially. The spillover of advanced management philosophies and technologies from foreign-invested companies also delivers demonstration effects on the host country. Companies at the host country would imitate and replicate the best practice, leading to productivity improvement at the host country. Second, a country can improve export product quality by optimizing market and business environment to attract more FDI [29,30]. As BRI countries improve their openness and business environment, FDI inflow will continue to grow and promote market competition. Under the huge pressure brought by FDI, domestic companies seek collaboration with well-established domestic counterparts to upgrade technologies and enhance their competitiveness. Market competition and productivity improvement decrease the product price under the same category. Technological progress maintains product quality at a reasonable range, further improving the competitiveness of export intermediate products. Quality improvement of export products represents the core competitiveness for companies to move up on the global value chain, making themselves fully embedded into the global value chain and improving their status. Overall, in terms of the sub-indicators of business environment, deregulation, enhancing intellectual property rights protection, and improving cross-border trade facilitation help attract FDI inflow, optimize industrial mix, and promote high-quality development. Fair and just factor market at the mature market economy environment can attract FDI inflow and relax financing restrains, offering more capitals for companies in R&D [31]. Improving the innovation capability of manufacturing industry can accelerate its upgrading and transformation and contribute to quality development [32], which will ultimately elevate the country’s status on the global value chain. This study therefore proposes the third hypothesis.

**Hypothesis** **3.** *Improvement in business environment elevates, by attracting FDI inflow, BRI countries’ status on the global value chain*.

## 3. Data and Statistical Model 

We incorporate business environment, FDI, and status elevation on the global value chain into a unified analysis framework to examine the impact of business environment and FDI on status elevation on the global value chain.

### 3.1. Data

Data employed in this study come from three sources. The first is the business environment data of 190 economies from *Doing Business Project* by the World Bank (Washington, DC, USA). We sift out 134 countries involved in the Belt and Road Initiative. However, due to availability of data, only 112 BRI countries are selected as the research subject and their characteristic variables are controlled. The second type of data are control variable data from *Worldwide Governance Indicators* and *World Development Indicators* by the World Bank. The third type is trade data from UN Comtrade Database. The five-digit category data of the Standard International Trade Classification Revision 3 (SITC Rev.3) which covers 2780 types of commodities are used for the calculation of export technology sophistication. FDI data are from database of the United Nations Conference on Trade and Development (UNCTAD). This study adopts the panel data of 112 countries from 2007 to 2017 to investigate the impact of FDI and business environment on the status elevation on the global value chain.

### 3.2. Variables

1. Dependent variable: BRI countries’ status on the global value chain (GVCs). Koopman (2012) decomposes the total exports of China and the U.S. to measure the status of manufacturing industries on the global value chain [33]. This study adopts sophistication level of exports, coined by Hausmann et al. (2007) [34], as the substitution indicator for status on the global value chain. However, according to the methods by Schott (2008), export similarity index among two countries can be measured by the following equation [35]:
  (1) $$ESI_{i}=\sum_{j}Min(S_{j,i},S_{j,r})$$
  where *j* represents the export product, *r* is the reference country and *i* is the sample country. $S_{j,r}$ is the share of product *j* in the total export of country *r*. *Min* refers to the smallest number in an interval. If the export in a sample country showcases a high degree of similarity with a developed country with advanced technologies, it means the sophistication level of export in the sample country is relatively high. In other words, the sample country holds a relatively high status on the global value chain. To minimize the impact of intermediate product import, this study constructs the net export similarity index among two countries as follows.
  (2) $$NESI_{i}\sum_{j}min\left(\frac{NE_{j,i}}{\sum_{j}NE_{j,i}},\frac{NE_{j,r}}{\sum_{j}NE_{j,r}}\right),\;\mathrm{and}\;NE_{j,i}=\{\begin{array}{c} E_{j,i}-M_{j,i},\;if\;j\in B\\ E_{j,i},if\;other \end{array}$$

In Equation (2), *E_j,i_* denotes the export value of product *j* in country *i*, *M_j,i_* is the share of product *j* import, *B* represents the set of intermediate products. According to the classification of broad economic categories by the United Nations, trade products are divided into primary products, intermediate products, and final products. Intermediate products include semi-finished products and parts and components. Based on the five-digit product category of the Standard International Trade Classification (SITC Rev.3) and BEC, this study makes the following treatment while calculating *NESI*. If product *j* is an intermediate product. It will be calculated by its net export value. If its net export value is negative, the observation will be scored as 0, meaning country *i* is incapable of export this kind of intermediate product; if product *j* is not an intermediate product, its export value will just be calculated by its total export value. The value of *NESI* falls into the range [0,1]. The bigger the value, the higher the similarity among the two countries in export structure and sophistication level of export, and the higher their status on the global value chain. When two countries export completely different products, the value of *NESI* is 0. On the other way round, its value is 1. For the convenience in comparison, this study adopts the average coefficients of net export similarity between Germany, the U.S., and Japan as the reference value to examine the evolution of and difference in sophistication level of export of BRI countries and to measure their manufacturing industries’ status on the global value chain. The calculation results based on the data of 112 BRI economies from 2007 to 2017 demonstrate that *NESI* of developed countries are generally higher than the others. *NESI* of China continues to grow with each passing year.

2. Key independent variables: (1) business environment (dtf). Business environment is an important indication of economic soft power of a country. Premier Li Keqiong once noted that business environment is in itself productivity. Business environment in China has been improving on the whole. According to *Doing Business 2019 Report* by the World Bank, business environment in China was ranked 46, elevated by 32 places compared with 2018. Significant progress has also been made in multiple sub-indicators of business environment. Electricity indicator improved by 84 places, starting businesses indicator grew by 65 places, and protection for minority investors increased by 55 places. Djankovetal (2010) attests that the indicator representing business environment of a country in the ease of doing business index by the World Bank is consistent with theories about FDI [36]. Pinheiro-Alves and Zambujal-Oliveira (2012) demonstrate the efficacy of the ease of doing business index in interpreting business environment through factor analysis and Cronbach’s alpha [37]. Referring to Li (2018), this study adopts ease of doing business score to measure the overall business environment of an economy [38]. Five sub-category indicators are used to investigate the specific category of business environment, including facilitation for construction permission (construct), facilitation for paying taxes (tax), facilitation for protection over investors (protect), facilitation for contract enforcement (contract), and facilitation for insolvency. The five sub-indicators accurately reflect the specific changes in business environment at each economy over time. The impact of business environment change on economies can also be detected in an accurate manner. The higher the business environment indicator, the more convenient the conditions for operation activities in the country. (2) foreign direct investment (FDI). In recent years, thanks to continuous efforts in opening-up and consistent improvement in business environment, the use of FDI in China has been kept in a good momentum, forming a stark contrast with the downward trend of FDI globally. China ranked third, behind the U.S. and the U.K., in terms of FDI use in 2016. One year later, China secured the second place in FDI use. According to the statistics of United Nations Conference on Trade and Development (UNCTAD), global FDI experienced a four-year downturn with a decline of 31.5% in 2019 from the 2015 level. In 2019, the total FDI in China, with banks, securities, insurances, and other fields included, reached 141.2 billion USD, increased by 2.1% over the preceding year. China’s share of FDI in global FDI grew from 6.7% in 2015 to 10.1% in 2019, up by 3.4 percentage points. Tang and Zhang (2017) reveals FDI inflow boosts inflow of foreign intermediate products and introduction of advanced technologies, which improves quality of export products, increases the domestic value added in gross export, and ultimately elevates the status on the global value chain [19]. However, FDI may also impede the status elevation on the global value chain due to the competition brought by imported intermediate products and lock-in on low end of the value chain. This study adopts FDI stock of countries to measure FDI level at different countries. Data are from the database of UNCTAD.
3. Control variables. In reference to existing research, this study selects the following control variables which relates to BRI countries’ status on the global value chain. (1) export scale. This study uses the logarithm of the export value in the database of World Development Indicators by the World Bank; (2) resource endowment. The share of the sum of historical export of ores, metals, and fuels in GDP is adopted as its substitution variable; (3) intellectual property rights protection. It is measured by the intellectual property right payment with relevant data derived from World Development Indicators (WDI) of the World Bank; (4) interest rate. High interest rate affects return on investment and investment cost of multinationals, which is a vital impacting factor for FDI. Data are from WDI; (5) per capita wealth (wealth). It is denoted by the logarithm of per capita GDP, and data come from WDI; (6) domestic average level of production (level). It is denoted by the ratio of value added of industry to sales value of industry. Data are from WDI; (7) industrial openness (open). It is measured by the ratio of trade volume to sales value of industry with data derived from WDI. The descriptive statistics of key variables are shown in Table 1.

### 3.3. Econometric Model

Based on the above analysis, this study starts by constructing the econometric model on the impact of business environment on BRI countries’ status elevation on the global value chain.

(3) $$lnGVCs_{it}=\alpha +\beta lndtf_{it}+\delta X_{it}+\gamma_{t}+\epsilon_{it}$$

where *i* refers to the country and *t* represents the year. Dependent variable *GVCs* denotes BRI countries’ status on the global value chain. Independent variable *dtf* is business environment, *X_it_* is the vector of relevant control variables, $\gamma_{t}$ is the year fixed effect. $\epsilon_{it}$ is the stochastic disturbance term.

In addition, to identify the impact of FDI on the status on the global value chain, we add FDI variable into Equation (3) and obtain the following equation.

(4) $$lnGVCs_{it}=\alpha +\beta lndtf_{it}+\varphi lnFDI_{it}+\delta X_{it}+\gamma_{t}+\epsilon_{it}$$

Furthermore, this study adds the interaction between business environment and FDI into Equation (4) to investigate the role of business environment in FDI’s impact on the statue on the global value chain. The specific model is shown below.

(5) $$lnGVCs_{it}=\alpha +\beta lndtf_{it}+\varphi lnFDI_{it}+\tau lndtf_{it}*FDI_{it}+\delta X_{it}+\gamma_{t}+\epsilon_{it}$$

This study also divides industries, based on their production factor, into labor-intensive industry, resource intensive industry, capital intensive industry, and technology intensive industry to explore the heterogeneity of the impact of business environment on the statue on the global value chain due to factor difference among industries. The model is constructed as follows:

(6) $$lnGVCs_{it}=\theta_{1}zy_{it}+\theta_{2}zb_{it}+\theta_{3}ld_{it}+\theta_{4}js_{it}$$

where *zy* denotes resource intensive industry. *zb* is resource intensive industry, *ld* represents labor-intensive industry, *js* represents technology intensive industry.

## 4. Empirical Results

### 4.1. Benchmark Regression Results

We first conduct Hausman tests to identify model setting. The Hausman test result shows that *p* = 0.0000, so the null hypothesis is rejected, which is indicating the fixed-effects model is preferred. Therefore, unless specifically noted, a fixed-effects model is employed in the following sections. We first analyze the relation between business environment and BRI countries’ status on the global value chain. Model (1) is the benchmark model for the impact of business environment on the status on the global value chain. Model (2) is constructed by adding the impact of foreign direct investment (FDI) into model (1). Model (3) is constructed by adding the interaction between business environment (dtf) and foreign direct investment into model (2), which means model (3) considers whether spillover effect of business environment is restrained by FDI. The results are presented in Table 2.

Empirical results reveal when FDI is not taken into consideration the coefficient of business environment in model (1) is 8.2060, which is significant at the 1% level. Once percentage point increase in business environment elevates the status on the global value chain by 8.2060 on average. This demonstrates business environment delivers positive effects on status elevation on the global value chain. One possible explanation is that favorable business environment lowers, to a large extent, entry costs and operation costs, which reduces investment risks, improves expectation on investment benefits, and is beneficial to independent innovation of companies. Therefore, Hypothesis 1 is proved. The impact of FDI and business environment on status elevation on the global value chain is considered in model (2). The empirical results show the estimate coefficient of FDI, and business environment is positive at the 1% significance level, meaning improvement of business environment and promotion of FDI contribute to elevation on the global value chain. The main reason is that FDI enables technological progress and innovation, improves the quality of export product, and increases the value added of export product, leading to status elevation on the global value chain. Thus, Hypothesis 2 is demonstrated. Results of model (3) show, when the interaction between business environment and FDI is added, the coefficient of business environment, FDI, and the interaction is 6.2188, 1.8610, and 3.1334 respectively, all of which are significant at the 1% level. The results indicate the improvement of the business environment can strengthen the positive effect of FDI inflows on the BRI countries’ global value chain upgrading.

To corroborate the above-mentioned mechanisms, this study further performs an intermediate model (Baron and Kenny, 1986) to test how the improvement of business environment attracts FDI to promote BRI countries’ global value chain upgrading [39]. We choose foreign direct investment (FDI) as mediators. We follow their method in four steps (Baron and Kenny, 1986). In the first step, we run the regression of business environment on the BRI countries’ global value chain upgrading to calculate the total effect. In the second step, we show the correlation between mediators and the business environment by running regression models of mediators on the business environment, individually. In the third step, we include both the business environment and mediators in a regression. By doing the second and third steps, we can successfully separate the direct effect of the business environment on BRI countries’ global value chain upgrading from the indirect intermediate effect. If both the business environment and mediator variables in the third step are significant, then the mediator is a partial transmission channel for the impact of the business environment on BRI countries’ global value chain upgrading. If only the mediators are significant in the third step, then it is a complete transmission channel. The results are shown in detail in Table 3.

The Sobel test can be used to judge the significance of the intermediate effects. The *p* value of Sobel test is close to 0, demonstrating the significance of FDI’s intermediate effects. The estimate results of main independent variables show optimizing business environment delivers positive effects on elevation on the global value chain at a significant level. FDI promotes status elevation on the global value chain at the 1% significance level. Such results demonstrate FDI’s intermediate effects are significant with the value being 2.9604 (2.7787 × 1.0654). The estimate results of FDI’s intermediate effects reveal FDI inflow promotes, to some extent, status elevation on the global value chain. Improvement of business environment means more favorable operation environment and system for foreign-invested companies, which lowers operation costs and risks. Increasing number of premium foreign companies and technologies is therefore channeled into the domestic market, which improves FDI quality, drives domestic technological innovation, and increases value added of export product. It is through improving FDI quality that progress in business environment promotes status elevation on the global value chain. Thus, Hypothesis 3 is proved. Last, the estimate results of control variables are consistent with those of the benchmark model, which are then not repeated here due to limitation of space.

### 4.2. Industry Heterogeneity Analysis

As shown in model (6), this study also divides the global value chain of different industries, based on the method of Wang (2017) [40], into labor-intensive industry (*ld*), resource intensive industry (*zy*), capital intensive industry (*zb*), and technology intensive industry (*js*) to explore the heterogeneity of the impact of business environment on the statue on the global value chain due to factor difference among industries. According to the empirical results, the estimate coefficients of business environment (dtf) are positive at the 1% significance level and their value is smaller than the coefficients in the benchmark model. The value change in the estimate coefficients of business environment demonstrate the impact of business environment on status elevation on the global value chain is heterogeneous among varied industries. Business environment significantly promotes status elevation on the global value chain for different industries. However, compared with the coefficient of labor-intensive industry, those of resource intensive industry and technology intensive industry are higher, meaning the effects of resource or technology intensive industry are stronger. Meanwhile, the coefficients of foreign direct investment (FDI) are positive at the 1% significance level for the four types of industries. FDI promotes status elevation on the global value chain for industries with varied factor intensity, and its promotion effects on resource intensive industry are the highest with its coefficient being 0.0416. The reason may be that resource intensive countries need to attract foreign direct investment for advanced and new technologies to promote resource exploitation and improve productivity. Similarly, FDI companies are the ones with advanced technologies and need to lower production costs through cheap labor, which in return increases domestic value added in gross export and elevates the status on the global value chain. Therefore, FDI also promotes status elevation for labor-intensive countries. As for technology intensive countries, FDI may enable integration and innovation of new technologies and therefore help them move up to the medium and high range on the global value chain. The results are shown in detail in Table 4.

### 4.3. Robustness Checks

Referring to Li (2018) [38], this study adopts the five sub-category indicators of business environment to conduct the robustness checks on the effects of business environment on BRI countries’ global value chain upgrading, including facilitation for construction permission (construct), facilitation for paying taxes (tax), facilitation for protection over investors (protect), facilitation for contract enforcement (contract), and facilitation for insolvency (insolvency). The five sub-indicators accurately reflect the specific changes in business environment at each economy over time. The higher the business environment indicator, the more convenient the conditions for operation activities in the country. The estimate results are shown in Table 5, which are basically consistent with the previous results, so Hypotheses 1 and 2 are further demonstrated.

## 5. Further Analysis

According to the above econometric test results, business environment and FDI significantly change the status on the global value chain. However, when the business environment and FDI are at different levels, their role in the promotion of the status of the BRI countries’ global value chain upgrading may be different, i.e., the role of the business environment and FDI in the promotion of the BRI countries’ global value chain upgrading may have a threshold effect. At this time, the traditional linear model assumes that the influence of the business environment and FDI on the BRI countries’ global value chain upgrading is fixed, so we need to re-design a model, when the independent variables are in different ranges, the impact on the dependent variable is different. Therefore, this study adopts business environment and FDI as the threshold variable respectively for threshold tests. The results are shown in Table 6 and Table 7.

Table 6 shows that the F value of the single threshold, double threshold, and triple threshold of FDI is 6.565, 197.688, and−0.921 respectively. The results of single threshold test and triple threshold test are significant at the 5% level while those of the double threshold test is positive at the 1% significance level. Therefore, these demonstrate business environment delivers significant double threshold effects on the status elevation on the global value chain with the estimate threshold value being 3.305 and 4.903, respectively. According to the empirical results of the panel threshold model, when FDI is lower than 3.305 the spillover effect of business environment is significant, and the elastic coefficient is positive at a statistically significant level. A 1% improvement in business environment leads to status elevation on the global value chain by 0.0595%. This indicates spillover of business environment significantly promotes status elevation on the global value chain. When FDI is higher than 3.305 and is lower than 4.903, business environment promotes status elevation on the global value chain more significantly. The elastic coefficient is 0.125. It is, therefore, identified that there is significant difference in the impact of business environment on status elevation on the global value chain at varied threshold ranges.

Generally, when FDI continues to increase, the positive impact of business environment on status elevation on the global value chain becomes increasingly significant. The main reason is that improvement in business environment promotes status elevation on the global value chain more significantly along with continuous FDI inflow. On the one hand, improving business environment makes capital registration more convenient and cuts costs and time. Companies can enter the market quickly, take preemptive actions, and seize beneficial export opportunities. On the other hand, improving business environment helps protect the legitimate rights and interests of investors and offers favorable and market-based financing conditions. Investors are therefore motivated, and more social capital can be mobilized, leading to rapid development, improvement of export product quality, technological innovation, and status elevation on the global value chain.

According to Table 6, F value of business environment (dtf) double threshold test is 102.379, respectively. which is significant at the 1% level. However, the single threshold test and triple threshold test is not statistically significant. Therefore, foreign direct investment (FDI) delivers double threshold effects on the status elevation on the global value chain. Its threshold value is 56.100 and 65.600, respectively. Based on the empirical results of the threshold model, when business environment (dtf) is below 41.800 the impact of FDI on the status elevation on the global value chain is not statistically significant with the elastic coefficient being 0.143. In another word, when business environment (dtf) is lower than 56.100, FDI does not promote status elevation on the global value chain. When business environment ranges from56.100 to 65.600, FDI’s spillover effect is still significant at the 1% level. The elastic coefficient is 0.774. When business environment is higher than 65.600, FDI promotes status elevation on the global value chain more significantly. A 1% increase in FDI elevates status on the global value chain by 1.338%. Therefore, FDI delivers varied promotion effects on status elevation on the global value chain in different threshold interval of business environment (dtf). Though FDI’s impact remains positive, its impact intensity is different.

The reasons for the above phenomenon are explained as follows. Along with the improvement of business environment, FDI’s promotion effect on status elevation on the global value chain becomes increasingly significant. Favorable business environment attracts large number of foreign companies to establish close cooperation ties through FDI. The integration and introduction of technologies improve product quality and productivity, leading to status elevation on the global value chain. When business environment reaches certain level, desirable conditions for business and legal support attract more foreign companies to increase investment. In such cases, FDI’s positive role in promoting technological innovation is in full play. While bringing in fierce competition, FDI also enables technological upgrading at local companies, helping companies move from the low end to the medium and high end of the global value chain.

## 6. Conclusions and Policy Implication

With the gradual expansion of the influence of the Belt and Road Initiative, BRI countries are also facing some new challenges, such as backflow of high-end industries and outflow of low-end industries. Solving the manufacturing bottleneck, enhancing the competitiveness of the international division of labor, and finally moving towards the mid to high end of the global value chain, has become an urgent problem for BRI countries. Therefore, this article attempts to discuss the issue of global value chain upgrading in the BRI countries from the perspective of business environment.

This study incorporates business environment, foreign direct investment (FDI), and the global value chain upgrading into a unified analysis framework to unravel the effects of business environment and FDI on BRI countries’ status elevation on the global value chain. The panel data of 112 BRI countries from 2007 to 2017 are employed for empirical tests on the trilateral relationship through the panel data regression model. The results show: (1) business environment improvement and FDI inflow significantly promote BRI countries’ status elevation on the global value chain. Business environment not only elevates BRI countries’ status on the global value chain directly but indirectly lifts their status through the intermediate effects of FDI; (2) business environment and FDI significantly promote the status elevation on the global value chain for industries that are intensive on varied factors, especially for labor-intensive industry; (3) the test results of the panel threshold model further verify the positive effect of the business environment and FDI inflows on BRI countries’ status elevation on the global value chain.

The conclusion of this study carries rich policy implications. New generation of information technology is fully integrated into manufacturing industry, triggering far-reaching industrial revolution. After the global financial crisis in 2008, there has been a trend of deglobalization at the world economy. Developed countries implemented reindustrialization and reshoring manufacturing strategies, which accelerates the formation of the new global trade and investment pattern. Although it is a good opportunity to move up the global value chain, evolution of the global value chain brings higher requirements for a business environment. BRI countries should focus on the following two aspects to construct the business environment which fits in the new situation of and changes in globalization to attract premium FDI inflow. First, the government of BRI countries should make more efforts in improving business environment to strengthen the supporting role of business environment in elevating the status on the global value chain. Efforts should be made in streamlining administrative approval system, deregulation, delegation of power to governments at lower levels, improvement of operational and post-operation oversight mechanism, improvement of government service efficiency, burden-easing for market entities, and improvement of public services. The government ought to improve business environment by resolving the problems for companies, enforcing favorable policies, and eliminating obstacle restraining companies’ development to strengthen their confidence and competitiveness. Second, the government should promote FDI inflow and give full play to the complementary role of business environment and FDI inflow.

## Figures and Tables

**Table 1:** Descriptive statistics of variables.

	Variable	Code	Mean	SD	Minimum	Maximum
Dependent variable	Status on global value chain	chain	8.207649	4.898577	0	23.34099
Independent variable	Business environment	dtf	5.893868	0.2909366	4.906891	6.483816
	Foreign direct investment	FDI	4.162892	0.9984578	−5	6.894549
Control variable	Per capita wealth	wealth	17.51375	14.57597	0	70.59955
	Intellectual property rights protection	iprp1	6.463159	3.167798	0	10.72787
	Resource endowment	resource	16.07034	26.81276	0	99.98649
	Industrial openness	open	85.21757	46.78791	0	437.3267
	Export scale	exscade1	9.50821	2.439018	0	12.35098
	Interest rate	rate	5.543384	37.4895	−31.9229	1158.026
	Domestic average level of production	Level	27.7054	12.73778	0	74.8123

**Table 2:** Business environment’s impact on BRI countries’ status elevation on the global value chain.

VARIABLES	(1) GVCs	(2) GVCs	(3) GVCs
dtf	8.2060 ***	6.8436 ***	6.2188 ***
	(16.195)	(13.885)	(13.276)
FDI		1.4064 ***	1.8610 ***
		(10.804)	(14.230)
Dtf * FDI			3.1334 ***
			(9.988)
wealth	0.0436 ***	0.0391 ***	0.0476 ***
	(4.498)	(4.315)	(5.548)
resource	0.0177 ***	0.0196 ***	0.0212 ***
	(3.090)	(3.645)	(4.178)
iprp1	0.4248 ***	0.3026 ***	0.2319 ***
	(8.207)	(6.081)	(4.894)
open	−0.0052 *	−0.0045 *	−0.0063 ***
	(−1.944)	(−1.825)	(−2.665)
exscade1	0.3426 ***	0.2657 ***	0.2133 ***
	(4.565)	(3.805)	(3.233)
rate	−0.0318 *	0.0134	0.0106
	(−1.920)	(0.850)	(0.713)
level	−0.0354 ***	−0.0576 ***	−0.0646 ***
	(−2.805)	(−4.863)	(−5.775)
Constant term	−44.9620 ***	−40.7077 ***	38.5919 ***
	(−16.053)	(−15.455)	(4.639)
Year fixed effect	YES	YES	YES
Observation	805	793	793
R-squared	0.556	0.620	0.663
Number of countries	112	112	112

Note: The t value in the parentheses is calculated using the prefecture-level clustering robust standard error. *YES* means corresponding fixed effect is controlled. ***/* indicates the significance at the 1%/10% levels, respectively.

**Table 3:** Estimate Results of FDI’s Intermediate Effect.

Dependent Variables	GVCs	FDI	GVCs
dtf	4.7046 ***	2.7787 ***	4.0875 ***
	(0.3227)	(0.2949)	(0.3273)
FDI			1.0654 ***
			(0.1171)

Note: The t value in the parentheses is calculated using the prefecture-level clustering robust standard error. *** indicates the significance at the 1% levels, respectively.

**Table 4:** Heterogeneity analysis.

VARIABLES	(1) *ld*	(2) *zy*	(3) *zb*	(4) *js*
dtf	0.1828 ***	0.2039 ***	0.1132 ***	0.2253 ***
	(12.835)	(13.442)	(8.294)	(12.091)
FDI1	0.0412 ***	0.0416 ***	0.0353 ***	0.0407 ***
	(10.950)	(10.397)	(9.792)	(8.270)
wealth	0.0019 ***	0.0000	0.0000	0.0010 ***
	(7.217)	(0.090)	(0.136)	(3.036)
resource	−0.0000	−0.0006 ***	−0.0003 **	−0.0005 ***
	(−0.098)	(−3.844)	(−2.176)	(−2.660)
iprp1	0.0069 ***	0.0092 ***	0.0051 ***	0.0089 ***
	(4.827)	(6.029)	(3.714)	(4.731)
open	0.0001	0.0001	−0.0003 ***	−0.0001
	(0.887)	(1.304)	(−4.957)	(−1.062)
exscade1	0.0063 ***	0.0066 ***	0.0074 ***	0.0104 ***
	(3.100)	(3.079)	(3.838)	(3.922)
rate	−0.0008 *	−0.0012 **	−0.0003	−0.0006
	(−1.751)	(−2.506)	(−0.680)	(−1.059)
level	−0.0009 ***	−0.0000	−0.0002	−0.0015 ***
	(−2.729)	(−0.059)	(−0.677)	(−3.443)
Constant term	−1.1744 ***	−1.3156 ***	−0.7652 ***	−1.3863 ***
	(−15.430)	(−16.232)	(−10.491)	(−13.922)
Year fixed effect	YES	YES	YES	YES
Observation	793	793	793	793
R-squared	0.632	0.599	0.434	0.548
Number of countries	112	112	112	112

Note: The t value in the parentheses is calculated using the prefecture-level clustering robust standard error. *YES* means corresponding fixed effect is controlled. ***/**/* indicates the significance at the 1%/5%/10% levels, respectively.

**Table 5:** Robustness checks.

VARIABLES	(1) GVCs	(2) *ld*	(3) *zy*	(4) *zb*	(5) *js*
FDI1	1.2641 ***	0.0403 ***	0.0421 ***	0.0290 ***	0.0359 ***
	(11.602)	(12.869)	(12.474)	(9.793)	(8.661)
construct	0.0085	0.0007 ***	0.0012 ***	0.0005 ***	0.0007 ***
	(1.550)	(4.393)	(7.066)	(3.211)	(3.188)
protect	0.0145 **	−0.0001	−0.0001	−0.0003 *	0.0005 **
	(2.370)	(−0.403)	(−0.701)	(−1.696)	(2.053)
tax	0.0117 **	0.0003 *	0.0003	0.0000	0.0009 ***
	(2.150)	(1.842)	(1.486)	(0.216)	(4.528)
insolvency	0.0824 ***	0.0026 ***	0.0025 ***	0.0023 ***	0.0029 ***
	(13.656)	(14.990)	(13.504)	(13.795)	(12.693)
contract	0.0564 ***	0.0012 ***	0.0013 ***	0.0012 ***	0.0017 ***
	(7.066)	(5.146)	(5.207)	(5.759)	(5.760)
Constant term	−8.7577 ***	−0.3272 ***	−0.3769 ***	−0.2390 ***	−0.3327 ***
	(−13.274)	(−17.270)	(−18.429)	(−13.330)	(−13.256)
Year fixed effect	YES	YES	YES	YES	YES
Observation	1103	1103	1103	1103	1103
R-squared	0.661	0.675	0.638	0.525	0.591
Number of countries	112	112	112	112	Number of countries

Note: The t value in the parentheses is calculated using the prefecture-level clustering robust standard error. *YES* means corresponding fixed effect is controlled. ***/**/* indicates the significance at the 1%/5%/10% levels, respectively.

**Table 6:** Threshold Test and estimation results.

Threshold Variable	Test	F Value	*p* Value
FDI	Single threshold	6.565 **	0.021
	Double threshold	197.688 ***	0.000
	Triple threshold	−0.921 **	0.020
Business environment	Single threshold	5.997	0.443
	Double threshold	102.379 ***	0.000
	Triple threshold	−48.971	1.000

Note: ***/**indicates the significance at the 1%/5% levels, respectively. *p* value and threshold value are obtained by 1000 times of Bootstrap sampling with replacement.

**Table 7:** Estimation results of threshold model.

Variable	Business Environment (dtf)	Foreign Direct Investment (FDI)
wealth	0.0149 *	0.0308 ***
	(1.94)	(2.93)
iprp1	0.402 ***	0.194 ***
	(10.92)	(3.52)
resource	0.0269 ***	0.0326 ***
	(6.38)	(5.78)
open	0.00284	−0.00320
	(1.00)	(−1.28)
exscade1	0.331 ***	0.365 ***
	(6.31)	(4.92)
rate	0.00908 **	−0.0148
	(2.27)	(−1.13)
level	−0.0499 ***	−0.0215
	(−5.27)	(−1.61)
Dtf (L ≤ r1)		0.0595 ***
		(3.75)
dtf (r1 < L ≤ r2)		0.125 ***
		(8.87)
Dtf (L > r2)		0.155 ***
		(11.05)
FDI (L ≤ r1)	0.776 ***	
	(6.34)	
FDI (r1 < L ≤ r2)	1.165 ***	
	(9.20)	
FDI (L > r2)	1.338 ***	
	(10.80)	
Threshold	Double threshold	Double threshold
Constant	−1.765 ***	−4.153 ***
	(−3.00)	(−4.58)

Note: The t value in the parentheses is calculated using the prefecture-level clustering robust standard error. ***/**/* indicates the significance at the 1%/5%/10% levels, respectively.

## Data Availability

Publicly available datasets were analyzed in this study. This data can be found here: http://data.worldbank.org.
